# Octopus‐derived antioxidant peptide protects against hydrogen peroxide‐induced oxidative stress in IEC‐6 cells

**DOI:** 10.1002/fsn3.3000

**Published:** 2022-07-27

**Authors:** Bo Peng, Bingna Cai, Jianyu Pan

**Affiliations:** ^1^ Guangdong Eco‐Engineering Polytechnic Guangzhou China; ^2^ State Key Laboratory of Organic Geochemistry, Guangdong Provincial Key Laboratory of Environmental Protection and Resources Utilization Guangzhou Institute of Geochemistry, Chinese Academy of Sciences Guangzhou China; ^3^ CAS Center for Excellence in Deep Earth Science Guangzhou China; ^4^ University of Chinese Academy of Sciences Beijing China; ^5^ Innovation Academy of South China Sea Ecology and Environmental Engineering (ISEE) Chinese Academy of Sciences Guangzhou China; ^6^ Key Laboratory of Tropical Marine Bio‐Resources and Ecology/Guangdong Key Laboratory of Marine Materia Medica South China Sea Institute of Oceanology, Chinese Academy of Sciences Guangzhou China; ^7^ Southern Marine Science and Engineering Guangdong Laboratory (Guangzhou) Guangzhou China

**Keywords:** antioxidant peptides, *Octopus vulgaris*, oxidative stress

## Abstract

This study aims to find antioxidant peptides from octopus protein hydrolyzates and verify the protective effects against H_2_O_2_‐induced oxidative stress in IEC‐6 cells. After the alcalase hydrolysate was ultrafiltrated, purified by Sephadex G‐25 gel fractionation and semipreparative reversed‐phase high‐performance liquid chromatography (RP‐HPLC), 16 peptides were identified, and chemically synthesized. In particular, the peptides AQNY, AMMLAW, FEGAW, GGAW, VDTVVCVW, and VVCLW showed better oxygen radical absorbance capacity (ORAC) and ABTS radical scavenging capacity. Among them, the smallest‐molecular‐weight peptide GGAW exhibited the best antioxidant activity. Furthermore, GGAW protected IEC‐6 cells from H_2_O_2_‐induced oxidative damage by significantly reducing the generation of reactive oxygen species (ROS), malondialdehyde (MDA), and lactate dehydrogenase (LDH), and increasing the activity of superoxide dismutase (SOD) and glutathione peroxidase (GSH‐PX), thereby improving cell viability. These results indicated that the peptide GGAW possessed the antioxidant capacity to prevent oxidative stress damage.

## Graphical abstract

Alcalase hydrolysate of octopus showed the highest antioxidant activity. The peptides containing Trp at the C‐terminus showed higher antioxidant activity. GGAW prevented H2O2‐induced oxidative damage in IEC‐6 cells.

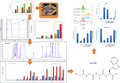

## 1 PREFACE

As natural antioxidants, food‐derived antioxidative peptides are considered to have high permeability and absorbability and confer various biological activities (Ghanbari, 2019). Scientific evidence has shown that antioxidative peptides prevent various chronic diseases associated with controlling oxidative stress (Sarmadi & Ismail, 2010). Hence, antioxidative peptides have been recommended for use as components in functional foods, cosmetics, and pharmaceuticals for health promotion.

Excessive production of reactive oxygen species (ROS) induced by oxidative stress damage is the main cause of intestinal diseases, such as gastroduodenal ulcers, inflammatory bowel disease, and colorectal cancer (Wang et al., 2021). Antioxidative peptides can scavenge ROS and free radicals by single‐electron and hydrogen transfer, and chelation of prooxidant transition metals (Zou et al., 2016). Antioxidative peptides show cellular antioxidant activity, reduce oxidative stress biomarkers, increase the activity of multiple antioxidant enzymes, and regulate the levels of antioxidant molecules (Aguilar‐Toala & Liceaga, 2020). It was reported that peptides (NPYVPR, AVPYPQR, KVLPVPEK, and ARHPHPHLSFM) are identified in milk‐protected Caco‐2 cells against oxidative stress induced by H_2_O_2_ by increasing the levels of both antioxidant molecules and antioxidant enzymes and reducing the production of ROS (Tonolo et al., 2018). In a similar study, Mirdamadi et al. found that three peptides (VLSTSFCPK, VLSTSFYPK, and STSFPPK) obtained from *Kluyveromyces marxianus* protein hydrolyzate could activate the Keap1‐Nrf2 signaling pathway, which may lead to a decrease in lipid and protein oxidation and cell apoptosis and an increase in cell viability (Mirdamadi et al., 2021).

*Octopus vulgaris* is an economically important seafood species worldwide because of its rich nutrition, strong environmental adaptability, fast growth, and high conversion rate (Luo et al., 2021; Vaz‐Pires et al., 2004). Octopus protein hydrolyzates obtained by treatment with various proteases were reported to exhibit antioxidant activities such as scavenging 1,1‐diphenyl‐2‐picrylhydrazyl (DPPH) radicals, preventing the bleaching of β‐carotene, and protecting DNA against breakage induced by hydroxyl radical (Slama‐Ben Salem et al., 2017). Tripeptide GEY was obtained from *Octopus aegina* mantle protein using gastrointestinal enzymes and possessed effective free radical scavenging in lipid peroxidation, DNA damage, and cellular destruction under stress conditions (Sudhakar & Nazeer, 2017). The alcalase hydrolyzate of *Octopus ocellatus* meat showed the highest scavenging effects against free radicals and hydrogen peroxide as well as the highest oxygen radical absorbance capacity and reduced the ROS production level in H_2_O_2_‐treated hepatocytes, without cytotoxicity (Um et al., 2017). However, the specific antioxidant peptide sequence from the octopus hydrolyzate and the antioxidant effects on oxidative stress remain to be evaluated.

In brief, the antioxidant activity of octopus hydrolyzates prepared with neutrase, alcalase, and papain was compared. Ultrafiltration, size‐exclusion chromatography, reversed‐phase high‐performance liquid chromatography (RP‐HPLC), and tandem mass spectrometry (MS/MS) were used for the separation, purification, and identification of the antioxidant peptides. Furthermore, the protective effects of the best antioxidant peptide on IEC‐6 cells subjected to H_2_O_2_‐induced oxidative damage were determined.

## 2 MATERIALS AND METHODS

### 2.1 Materials

Octopus was freshly obtained from the Huangsha aquatic products wholesale market (Guangzhou, China). Fresh octopus meat was separated, cut into small pieces, homogenized, and finally refrigerated (−20°C). Rat small intestinal epithelial cell‐line (IEC‐6) cells were purchased from American Type Culture Collection. Neutrase, alcalase, and papain were purchased from Pangbo Enzyme Co., Ltd. Fluorescein, DPPH, 2,2′‐azinobis‐3‐ethyl‐benzothiazoline‐6‐sulfonic acid (ABTS), 2,2′‐azobis(2‐methylpropion‐amidine) dihydrochloride (AAPH), 6‐hydroxy‐2,5,7,8‐tetrame thylchroman‐2‐carboxylic acid (Trolox), and 2′, 7′‐dichlorofluorescein diacetate (H2DCFDA) were obtained from Sigma Aldrich Co. All other chemical reagents used in the experiments were of analytical grade.

### 2.2 Preparation of octopus protein hydrolyzate

The octopus homogenate was mixed with twofold (g/ml) distilled water and then hydrolyzed separately using alcalase (55°C, pH 8.0), neutrase (50°C, pH 6.5), and papain (50°C, pH 6.5) at 3000 U/g for 5 h. The reaction was stopped by boiling the samples in a water bath at 100°C for 10 min. The hydrolyzates were centrifuged at 30,000 *g* (Avanti J‐26S XP Centrifuge, BECKMAN COULTER, Inc.) for 30 min. The supernatant was collected, concentrated, and freeze dried, resulting in the octopus protein hydrolyzate ON, OA, and OP from neutrase, alcalase, and papain hydrolysis, respectively. The protein content of hydrolyzate powder was determined according to the AOAC method. The degree of hydrolysis (DH) was measured by the o‐phthaldialdehyde (OPA) method described by Nielsen et al. (2001).

### 2.3 Determination of amino acid composition

The sample was hydrolyzed with 6 mol/L HCl in a nitrogen atmosphere at 110°C for 24 h. The amino acid composition was analyzed using a Hitachi 835‐50 automatic amino acid analyzer (Hitachi Co.) after hydrolysis.

The Trp level was measured after hydrolysis with 4 mol/L LiOH in a nitrogen atmosphere at 110°C for 20 h. The obtained hydrolyzates were filtered through a piece of filter membrane with a 0.22 μm pore size and determined by HPLC.

### 2.4 Analysis of antioxidant activity

The DPPH, ABTS assay, and ORAC values were conducted by the reported method (Agrawal et al., 2016; Wattanasiritham et al., 2016) with some modifications. Trolox solutions (100, 50, 25, 12.5, and 6.25 μmol/L) were used to establish the standard curve. The DPPH, ABTS activity, and ORAC values were shown as μmol TE/μg peptide using the standard curve established previously.

### 2.5 Isolation of antioxidant peptides

The octopus protein hydrolyzate OA, which showed the highest antioxidant activity, was further separated using ultrafiltration cut‐off membranes (Vivaflow 200, Sartorius), collecting five fractions with MWs >100 kDa, 10–100 kDa, 5–10 kDa, 3–5 kDa, and <3 kDa. The ultrafiltrates were vacuum‐concentrated, lyophilized, and then subjected to antioxidant assays.

The ultrafiltration component with the highest antioxidant activity was suspended in distilled water (30 mg/ml) and purified by a Sephadex G‐25 gel filtration. The gel column (300 × 45 mm) was eluted with distilled water using EZ Purifier III liquid chromatography (Shanghai Lisui Chemical Engineering Co., Ltd.) at a flow rate of 10 ml/min and monitored at 220 nm. The major peak was collected and lyophilized for antioxidant assay analysis and RP‐HPLC purification.

The fraction with strong antioxidant activity was then subjected to YMC‐Pack ODS‐A column (250 × 10 mm, I.D. S‐5 μm, 12 nm) on an Agilent 1260 system (Agilent Technologies) for further separation. The flow rate was maintained at 2.5 ml/min using eluent A (deionized water containing 0.1% trifluoroacetic acid, TFA) and eluent B (acetonitrile containing 0.1% TFA). The elution program was as follows: 1–10 min, 6% B; and 10–30 min, 6%–49% B. The isolated fractions were monitored at 220 nm. Eighteen fractions were collected, dried by nitrogen flow, and lyophilized for antioxidant activity assays.

### 2.6 Identification of peptide sequences

The fractionated peptides with the highest antioxidant activity were identified by high‐performance liquid chromatography–tandem mass spectrometry (HPLC–MS/MS) utilizing a Bruker Q‐TOF Premier mass spectrometer (Bruker Daltonic Inc.) coupled with electrospray ionization (ESI). The HPLC system was equipped with a YMC‐Pack ODS‐AQ (250 × 4.6 mm, 5 μm) column and used as follows: 1–10 min, 80% A (deionized water with 0.1% TFA), and 10–35 min, 20%–100% B (methanol with 0.1% TFA), at a flow rate of 1.0 ml/min. The ESI system was operated in positive mode with a capillary voltage of 3.8 kV and scan range of *m/z* 100–2000. The data were analyzed with PEAKS Studio software version 8.0 (Bioinformatics Solution Inc.). Only those peptides that presented “de novo” −10lgP above 20 and were similar to the protein sequences of octopus by BLAST were considered for further analysis.

### 2.7 Peptide synthesis

The biological peptides were synthesized using the FMOC solid‐phase procedure by ChinaPeptides Co., Ltd. The purity of the synthetic peptides was verified to be higher than 95% by analytical HPLC equipped with an API150‐ESI mass spectrometry system.

### 2.8 IEC‐6 cell‐line culture

High‐glucose Dulbecco's modified Eagle medium (DMEM) with 10% fetal bovine serum (FBS), 100 IU/ml penicillin and streptomycin, 2 mmol/L glutamine, 10 mmol/L 4‐(2‐hydroxyethyl)‐1‐piperazine ethane sulfonic acid (HEPES), and 10 μg/ml insulin were used for culturing IEC‐6 cells in a humidified incubator under 5% CO_2_ at 37°C. IEC‐6 cells were seeded at 2.5 × 10^5^ cells/well for 24 h. After treatment with 50, 100, and 200 μg/ml bioactive synthesized peptide for 24 h and then with 300 μmol/L H_2_O_2_ for another 6 h, the cell viability was determined by an MTT assay (Wang et al., 2021).

### 2.9 Detection of ROS, MDA, LDH, SOD, and GSH‐PX levels

IEC‐6 cells were seeded at 2.5 × 10^5^ cells/well after treatment as described in Section 2.8. Cells were harvested with trypsin and washed with PBS. Cellular ROS was detected by the probe fluorescein‐labeled dye H2DCFDA according to the method reported by Dai et al. (2020). The level of malondialdehyde (MDA), lactate dehydrogenase (LDH), superoxide dismutase (SOD), and glutathione peroxidase (GSH‐PX) contents were determined using the corresponding kits obtained from Nanjing Jiancheng Bioengineering Institute.

### 2.10 Statistical analysis

All data were expressed as means ± standard deviation (SD) and analyzed by one‐way analysis of variance (ANOVA) with the Bonferroni multiple‐range tests using IBM SPSS 22.0 software. *p* < .05 was considered as significant.

## 3 RESULTS AND DISCUSSION

### 3.1 Antioxidant activity of octopus protolyzate

**FIGURE 1:**
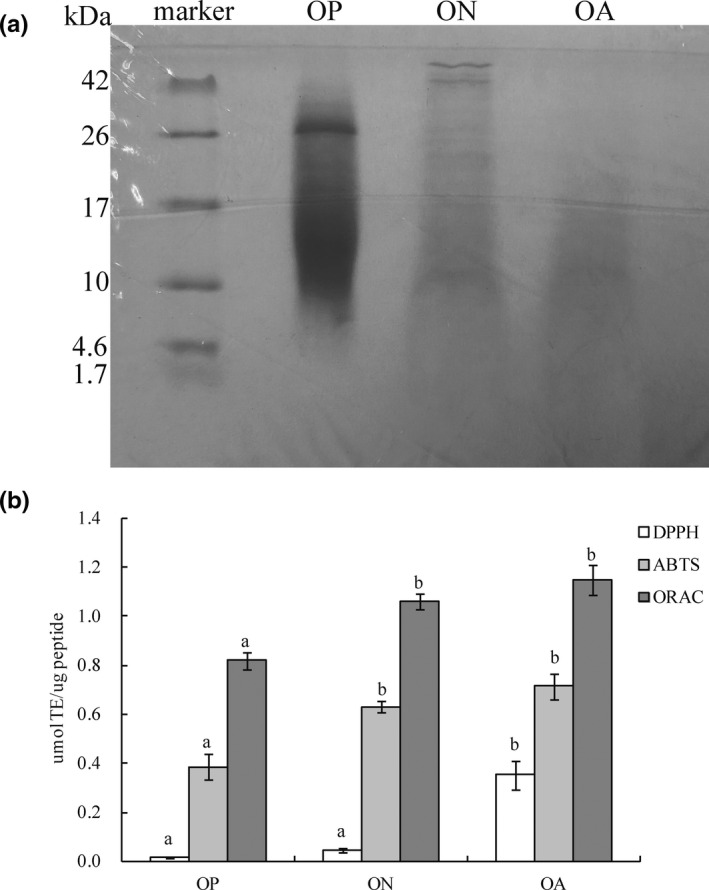
(a) Heatmap of the amino acid composition of each hydrolyzate and (b) antioxidant capacity of octopus protein hydrolyzate. Octopus protein hydrolyzate ON, OA, and OP was prepared by neutrase, alcalase, and papain hydrolysis, respectively. Different lowercase letters on the bar indicated significant differences (*p* < .05) between groups.

On the basis of the tricine SDS–PAGE results, the peptide pool composition of each hydrolyzate is reported in Figure 1a. The DH percentages of OA, ON, and OP after 5 h of incubation were 31.47 ± 0.78%, 30.70 ± 1.30%, and 26.87 ± 0.66%, respectively. Compared with the other hydrolyzates, OA, which had the highest DH and protein content, should be rich in low‐MW peptides. The DH affects the length and amino acid composition of peptides, which can greatly influence antioxidant activity (Sila & Bougatef, 2016). As reported in Table 1, the ON and OA hydrolyzates showed a similar amino acid composition, whereas OP displayed a different amino acid composition. Hydrophobic amino acids, Val, Ile, Phe, Lys, His, and Trp, frequently occurred in OA and ON, accounting for 26.21% and 26.20% of the total amino acids, respectively, whereas they were poorly expressed in OP (25.20%). In contrast, Glu, Pro, Gly, and Ala were the most abundant amino acid residues in OP. Moreover, the Fischer ratio of OA and ON was higher than that of OP, which was beneficial for higher radical scavenging activity (Lan et al., 2019).

**TABLE 1:** Composition of each hydrolyzate

	OA	ON	OP
Yield (%)	15.68	14.45	10.63
Protein (g/100 g)	83.11 ± 2.91	79.28 ± 3.32	73.36 ± 3.54
Amino acid composition (g/100 g)			
Asp	6.53	6.67	6.27
Thr	2.95	3.04	2.79
Ser	2.85	2.90	2.82
Glu	9.83	9.89	9.92
Pro	2.62	2.54	2.72
Gly	4.63	4.41	5.30
Ala	3.99	3.92	4.17
Val	3.13	3.13	3.01
Met	1.61	1.62	1.41
Ile	3.09	3.11	2.68
Leu	5.08	5.02	4.61
Tyr	0.84	1.03	0.89
Phe	3.93	3.77	3.75
Lys	4.61	4.69	4.54
His	1.20	1.11	0.92
Arg	5.55	4.93	4.83
Trp	0.55	0.51	0.51
HAA	16.51	16.32	15.41
Fischer ratio	2.86	2.86	2.70
Total	62.99	62.29	61.14

*Note*: Octopus protein hydrolyzates ON, OA, and OP were prepared by neutrase, alcalase, and papain hydrolysis, respectively. Yield (%) = (the weight of lyophilized hydrolyzate powder/the weight of wet octopus meat) × 100. Fischer ratio = (*M*_val_ + *M*_ile_ + *M*_leu_)/(*M*_Tyr_ + *M*_Phe_ + *M*_Trp_).

Abbreviation: HAA, hydrophobic amino acid including Ala, Tyr, Val, Ile, Leu, Phe, and Trp.

The antioxidant activity results of DPPH, ABTS, and ORAC assays, as shown in Figure 1b, demonstrated that OA possessed the highest radical‐scavenging abilities and potential antioxidant properties. These results are consistent with recent antioxidant studies that reported that the alkaline protease hydrolyzate from octopus protein exhibited the highest scavenging ability compared to octopus treated with other commercial enzymes (Um et al., 2017). Wang et al. (2021) reported that the DH was highly positively correlated with antioxidant activity. This finding also showed that the higher the DH value, the higher the antioxidant activity of OA, which contained more low‐molecular‐weight bioactive peptides. In addition, this result might be due to the more hydrophobic amino acids in OA. It was reported that peptides with high antioxidant activity contain a high proportion of hydrophobic amino acids, which is considered the key factor in the ability of peptides to scavenge radicals (Zou et al., 2016). Moreover, a significant difference in the DPPH radical scavenging ability between ON and OA was observed (*p* < .05). The content of Phe and His in OA, ON, and OP accounted for 8.14%, 7.83%, and 7.64% (Table 1), respectively. Mendis et al. suggested that the peptide showed higher radical scavenging activity when it contained more Phe and His residues (Mendis et al., 2005).

### 3.2 Isolation of antioxidant peptides

**FIGURE 2:**
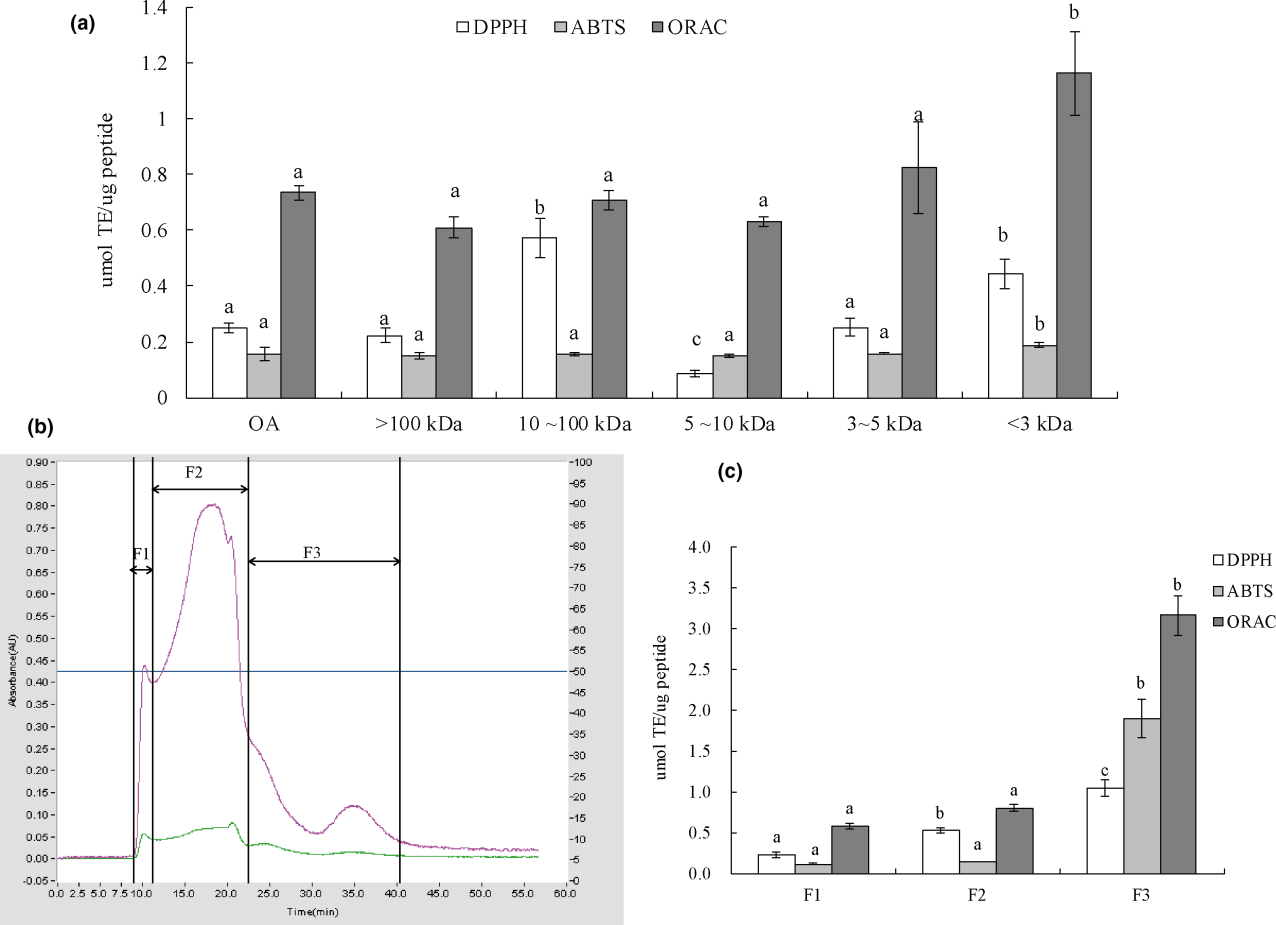
(a) The DPPH, ABTS^+·^, and ROO^·^ scavenging activities of fractions with different MWs. (b) Chromatogram profiles of separation of MW <3 kDa using Sephadex G‐25 gel at 280 nm (green line) and 220 nm (pink line). (c) The corresponding antioxidant activity of the separated gel fractions. Different lowercase letters on the bar indicated significant differences (*p* < .05) between groups.

The octopus protein was hydrolyzed by alcalase and subsequently ultrafiltrated. As seen in Figure 2a, the fraction with MW <3 kDa possessed higher DPPH, ABTS^+·^, and ROO^·^ radicals scavenging activity, with 0.44 ± 0.05 μmol TE/μg peptide, 0.19 ± 0.01 μmol TE/μg peptide, and 1.16 ± 0.15 μmol TE/μg peptide, respectively (*p* < .05). This result was consistent with previous research stating that protein hydrolyzates with low molecular weights have higher electron transfer efficiency and can scavenge radicals more effectively (Chalamaiah et al., 2012; Wen et al., 2020). After ultrafiltration, low‐molecular‐weight peptides were enriched, which exerted more effective antioxidant activity than the whole hydrolyzates.

The ultrafiltration fraction MW <3 kDa was further purified using Sephadex G‐25, as shown in Figure 2b, and three fractions with decreasing molecular weight were collected. The DPPH, ABTS^+·^, and ROO^·^ scavenging abilities of F3 were 1.05 ± 0.10 μmol TE/μg peptide, 1.89 ± 0.23 μmol TE/μg peptide, and 3.16 ± 0.24 μmol TE/μg peptide, respectively, which were significantly higher than those of F1 and F2 (*p* < .05; Figure 2c). The last fraction F3 with the smallest molecular weight peptides and free amino acids exhibited the highest DPPH, ABTS^+·^, and ROO^·^ scavenging activity. These results were similar to the reports that separated antioxidant peptides using Sephadex G‐25, and they both determined that the peptides in the last fractions presented the highest antioxidant activity (Jia et al., 2020; Zhang et al., 2018). Therefore, the F3 fraction was recommended as the major active component to be further investigated.

**FIGURE 3:**
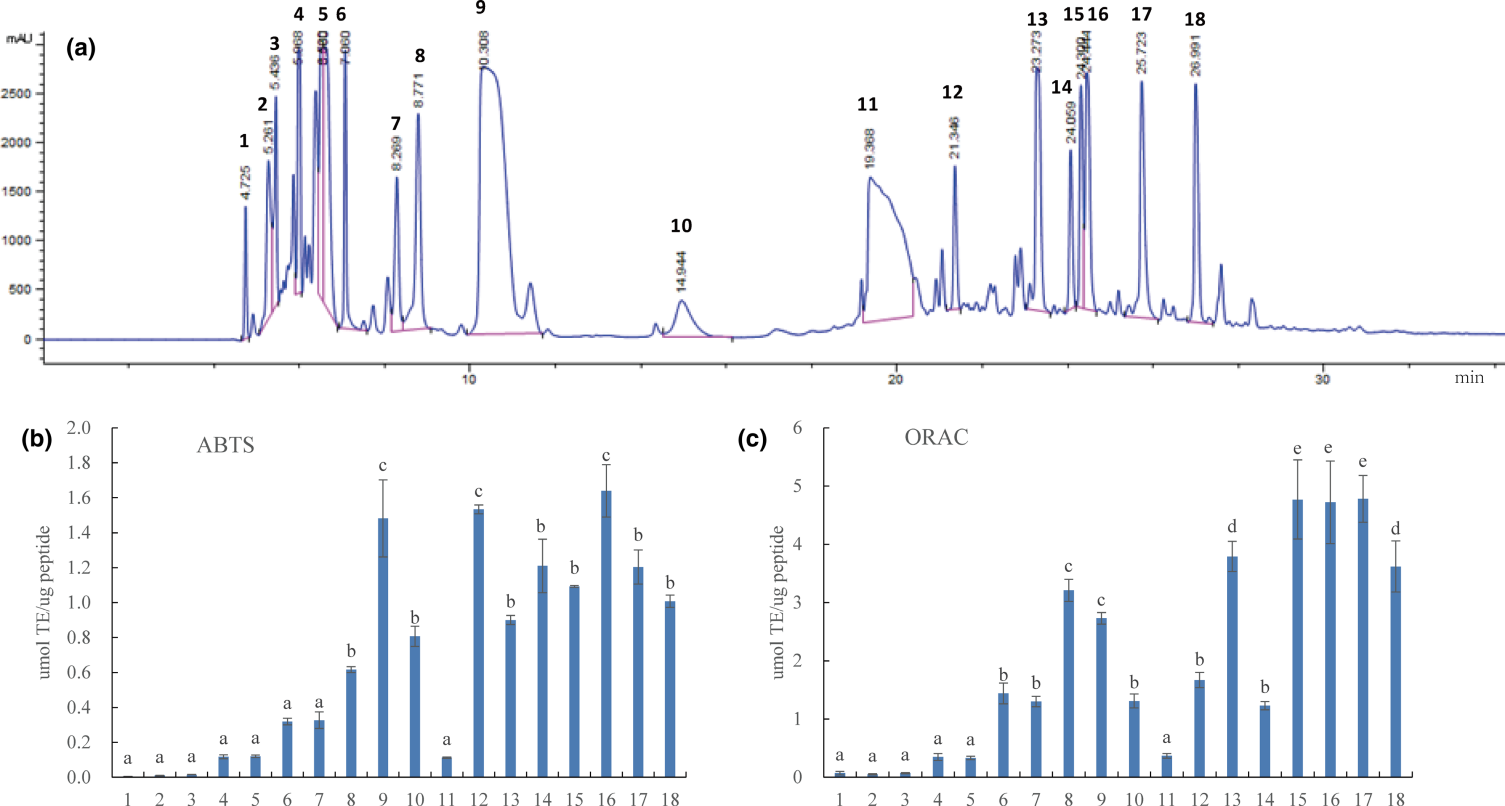
RP‐HPLC separation of F3 (a) with the corresponding scavenging activity of ABTS^+·^ (b) and ROO^·^ radicals (c). Different lowercase letters on the bar indicated significant differences (*p* < .05) between groups.

Next, RP‐HPLC was used to further separate the F3 fraction (Figure 3a). Fraction F3 was separated into 18 peaks; the peaks 15#, 16#, and 17# were found to exhibit higher antioxidant activities than those of the other peaks (Figure 3b,c). Many studies have shown that antioxidant peptides are mainly affected by amino acid composition, sequence, structure, and hydrophobicity (Wen et al., 2020). Thus, the amino acid sequences in fractions 15#, 16#, and 17# were identified using HPLC–ESI–MS/MS.

### 3.3 Sequence and activities of the antioxidant peptides

The 16 peptides recognized from fractions 15#, 16#, and 17# are summarized in Table 2. The characterized peptides consisted of 4–9 amino acid residues and had molecular weights between 389.17 and 919.45 Da. All the peptides were chemically synthesized to evaluate their ABTS^+·^ and ROO^·^ scavenging activity. As shown in Table 2, the peptide GGAW exhibited the highest ABTS scavenging ability and ORAC value, at 1.95 ± 0.16 and 5.37 ± 0.15 μmol TE/μg peptide, respectively.

**TABLE 2:** Peptides identified in fractions 15#, 16#, and 17# and their antioxidant activity.

Fraction	Peptide sequence	m/z	Mass (Da)	PeptideRanker score^a^	Hydrophobic^b^ (%)	ABTS (μmol TE/μg peptide)	ORAC (μmol TE/μg peptide)
15#	YDADPR	368.67	735.32	0.4985	33.33	0.11 ± 0.01	1.67 ± 0.04
	FFMR	600.30	599.29	0.9914	75.00	0.00 ± 0.00	1.70 ± 0.01
	DAERPK	358.19	714.37	0.1780	33.33	0.00 ± 0.00	0.00 ± 0.00
16#	AQNY	495.20	494.21	0.2200	25.00	0.24 ± 0.02	2.99 ± 0.03
	FKDDFL	392.71	783.38	0.8341	50.00	0.00 ± 0.00	0.00 ± 0.00
	AMMLAW	377.67	753.32	0.8906	100.00	0.26 ± 0.04	2.76 ± 0.08
	FEGAW	609.27	608.26	0.8630	60.00	1.16 ± 0.15	2.75 ± 0.01
	GGAW	390.18	389.17	0.9382	50.00	1.95 ± 0.16	5.37 ± 0.15
	FETTADA	377.66	753.32	0.0747	42.86	0.00 ± 0.00	0.04 ± 0.01
	AGMLVMM	392.67	783.33	0.8017	85.71	0.00 ± 0.00	0.95 ± 0.11
17#	VDTVVCVW	460.74	919.45	0.2870	62.50	0.81 ± 0.09	2.59 ± 0.05
	TTTVTT	623.32	622.32	0.0215	16.67	0.00 ± 0.00	0.00 ± 0.00
	VVTTEA	619.33	618.32	0.0233	50.00	0.00 ± 0.00	0.11 ± 0.04
	VVCLW	619.33	618.32	0.6665	80.00	1.37 ± 0.12	2.10 ± 0.10
	LKTT	462.29	461.28	0.0457	25.00	0.00 ± 0.00	0.03 ± 0.00
	SSEVVV	619.33	618.32	0.0708	50.00	0.00 ± 0.00	0.00 ± 0.00

^a^ According to “PeptideRanker” (PeptideRanker (ucd.ie)).

^b^ According to “Peptide2.0”(Custom Peptide Synthesis (peptide2.com)).

Structure–function relationships suggested that the antioxidant activity of peptides was more related to the hydrophobic and antioxidant amino acids (including Trp, His, Met, Phe, Tyr, Ala, Pro, Leu, and Gly) in the sequence as well as their molecular structures (Chen et al., 2021; Sila & Bougatef, 2016). It has been noted that the presence of hydrophobic amino acids, such as Ala, Trp, Tyr, Val, Met, Ile, Leu, and Phe in the antioxidant peptides could augment the interaction with radical species (Tkaczewska et al., 2019). The hydrophobic peptides FFMR, AMMLAW, FEGAW, GGAW, VDTVVCVW, and VVCLW (hydrophobic residues ≥50%) showed high antioxidant activity, most likely due to the hydrophobic amino acid content. It has also been reported that the peptides containing Trp at the C‐terminus showed stronger antioxidant activity, which could be due to the aromatic group stabilizing radicals through resonance or delocalization (Ghassem et al., 2017; Sila & Bougatef, 2016). As shown in Table 2, the peptide segments GGAW, AMMLAW, FEGAW, VDTVVCVW, and VVCLW with the presence of Trp at the C‐terminus form the strongest conventional hydrogen bonds, which contribute to enhancing their antioxidant activity. In addition, previous studies demonstrated that Gly is a potential target site of free radicals due to the presence of only a hydrogen atom in its side chain (Wu et al., 2018; Yang et al., 2020). In particular, peptides containing Gly are more flexible in exposing the functional residues to free radicals, causing a significant increase in antioxidant activity (Wu et al., 2021; Zhong et al., 2021). The antioxidant activity of GGAW was strongly attributed to its sequence containing 50% hydrophobic amino acids, two Gly residues at the N‐terminus, and Trp at the C‐terminal position.

### 3.4 Protective effects of GGAW against oxidative stress in IEC‐6 cells

**FIGURE 4:**
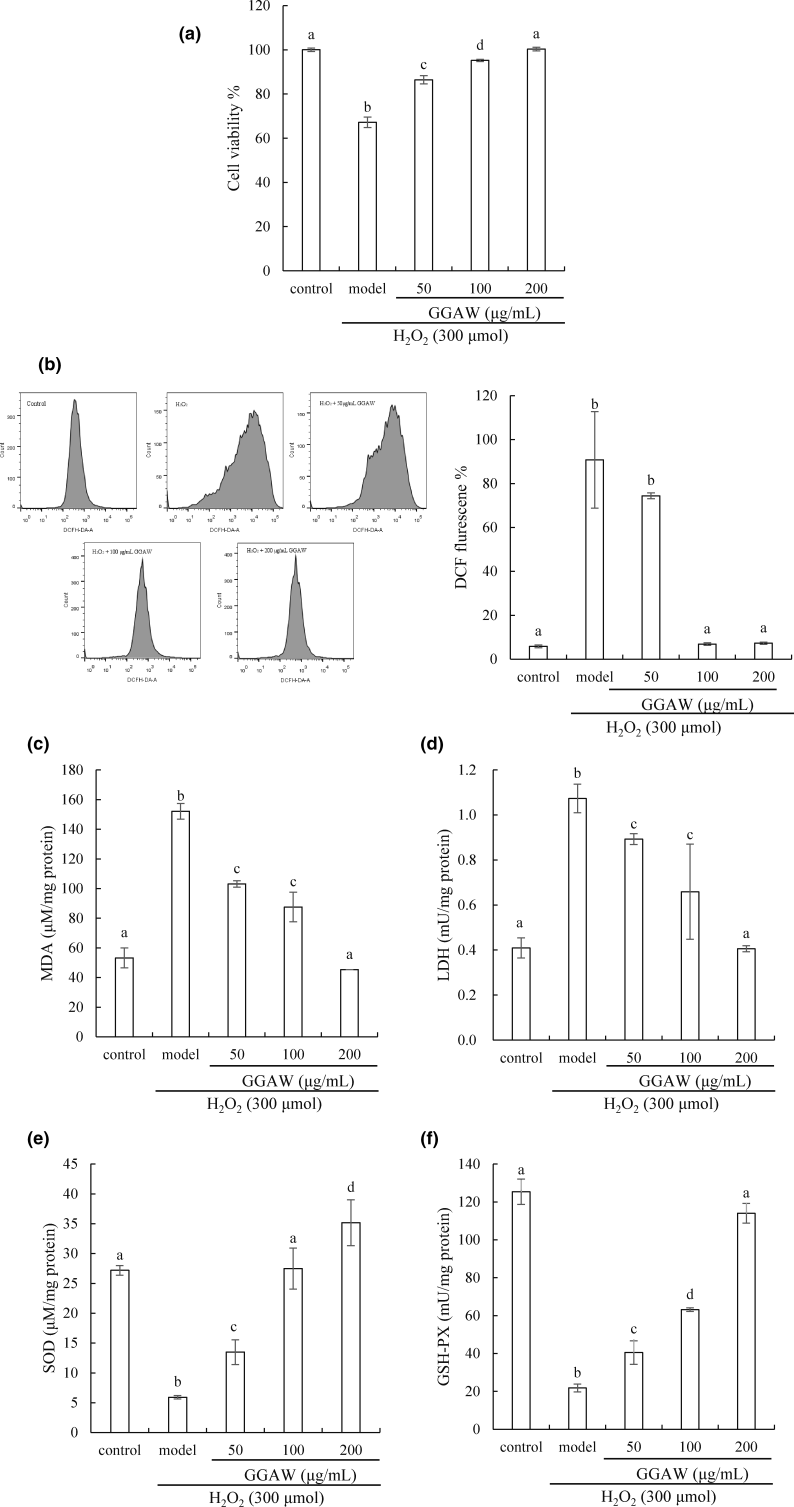
IEC‐6 cells were treated to 300 μmol/L H_2_O_2_ for 6 h and pretreated with GGAW at concentrations of 50, 100, and 200 μg/ml for 24 h. The effect of GGAW on cell viability was determined by CCK‐8 assay (a). Effect of GGAW on reactive oxygen species (ROS, b), malondialdehyde (MDA, c), lactate dehydrogenase (LDH, d), superoxide dismutase (SOD, e), and glutathione peroxidase (GSH‐PX, f) levels. Different lowercase letters on the bar indicated significant differences (*p* < .05) between groups.

The H_2_O_2_‐induced IEC‐6 cell oxidative damage model was used to study the protective effect of GGAW. The changes in cell viability after exposure to GGAW pretreatment for 24 h and 300 μmol/L H_2_O_2_ for 6 h are shown in Figure 4a. After exposure to H_2_O_2_ stimulation, cell viability was significantly reduced to 67.18 ± 2.39%, but GGAW pretreatment obviously improved cell viability in a dose‐dependent manner (*p* < .05). In particular, compared with the control group, the GGAW group had no significant difference in cell viability at a concentration of 200 μg/ml (*p* > .05). The results indicated that GGAW had effectively reversed the decline in cell viability induced by H_2_O_2_.

Hydrogen peroxide can rapidly diffuse through cell membranes and trigger oxidative stress, producing large amounts of ROS, which lead to cell damage (Di Marzo et al., 2018). Excessive ROS attack the polyunsaturated fatty acids in biofilms to form the lipid peroxidation product MDA. The higher the MDA content, the more severe the cell oxidative damage (Bouzenna et al., 2017). When the cell membrane is damaged, LDH is released into the blood (Wiriyaphan et al., 2015). As shown in Figure 4b, IEC‐6 cells treated with H_2_O_2_ alone significantly produced excessive ROS. However, exposure of IEC‐6 cells to various GGAW concentrations as pretreatment plus H_2_O_2_ resulted in a significant reduction in ROS levels (*p* < .05). Moreover, there was no significant difference in the ROS level in IEC‐6 cells treated with 100 and 200 μg/ml GGAW and in the control group (*p* > .05). It has been reported that GSH can protect epithelial cells from H_2_O_2_‐mediated oxidative stress, and 10 mmol/L GSH can significantly reduce cellular ROS levels (Ren et al., 2018). In this study, GGAW played positive protection at a lower concentration and may have better antioxidant activity and cytoprotection than GSH. The increase in ROS levels caused by H_2_O_2_ induced a significant increase in the MDA and LDH contents (Figure 4c,d), which reflected oxidative damage to the cell membrane. When IEC‐6 cells were cultured with GGAW at concentrations of 50–200 μg/ml, the generation of MDA and LDH was significantly inhibited in a dose‐dependent manner (*p* < .05). In particular, after pretreatment with 200 μg/ml GGAW, MDA and LDH generations were significantly decreased, with a significant difference from the control group (*p* < .05). Similar results were found with the peptide IRW, and its protective effect against oxidative stress was attributed to Trp, which can inhibit intracellular ROS accumulation and block the ROS‐activated mitochondria‐mediated cell apoptosis pathway (Yi et al., 2017). These results indicated that GGAW effectively weakened the oxidative damage induced by inhibiting ROS accumulation.

In addition, intracellular antioxidases such as SOD and GSH‐PX constitute the antioxidant defense system to eliminate excess ROS and combat and prevent oxidative stress in cells (Wang et al., 2016). SOD catalyzes the dismutation of two superoxide molecules to form molecular oxygen and hydrogen peroxide which are further converted into harmless substances by other enzymes, such as catalase and peroxidases (Nordberg & Arner, 2001). As shown in Figure 4e,f, after treatment with H_2_O_2_ for 6 h, the levels of GSH‐PX and SOD were significantly decreased compared with those of the control group (*p* < .05). However, when IEC‐6 cells under oxidative stress induced by H_2_O_2_ were pretreated with GGAW at a concentration of 50–200 μg/ml, the SOD activity was significantly enhanced compared with that of the model group (*p* < .05). In particular, the SOD content was higher at a concentration of 200 μg/ml GGAW than that of the control group (*p* < .05). The level of GSH‐PX was increased at a concentration of 200 μg/ml GGAW and was not significantly different from that in the control group (*p* > .05). These results showed that GGAW enhanced the antioxidant enzyme activity to maintain redox homeostasis and impair the cellular antioxidant systems. The cytoprotection of GGAW against H_2_O_2_‐induced injury may be due to its aromatic residue Trp and hydrophobic amino acids which possessed strong antioxidant properties. Trp‐containing peptides display a good ability to scavenge free radicals due to the existence of indole groups in Trp, which can better provide H^+^ to radicals. Hydrophobic amino acids Ala and Gly can provide H^+^ and enhance synergy with other amino acids to display antioxidant properties (Li et al., 2021). Previously studies have showed that some similar peptides, such as MKAVCFSL (Zhang et al., 2021), IYVFVR (Zhang et al., 2019), and WVSPLAGRT (Bollati et al., 2022), contained more aromatic and hydrophobic amino acids and efficiently ameliorated the damage of oxidative stress.

## 4 CONCLUSIONS

In this study, the octopus hydrolyzate prepared with alcalase showed higher antioxidant activity than the neutrase and papain hydrolyzates. After ultrafiltration and purification by Sephadex G‐25 and RP‐HPLC, 16 peptides were identified from the octopus alcalase hydrolyzate. Among them, AQNY, AMMLAW, FEGAW, GGAW, VDTVVCVW, and VVCLW showed better oxygen radical absorbance capacity and ABTS radical scavenging capacity. In particular, GGAW exhibited the strongest antioxidant activity due to its low molecular weight and the presence of 50% hydrophobic amino acids, antioxidant amino acid Trp at the C‐terminus, and Gly‐Gly at the N‐terminus. Furthermore, GGAW prevented oxidative stress induced by H_2_O_2_ by reducing the contents of ROS, MDA, and LDH while enhancing the activities of the intracellular antioxidant enzymes SOD and GSH‐PX. These results indicate that GGAW has great potential to be developed into functional foods due to its antioxidant activity.

## CONFLICT OF INTEREST

The authors declare that they have no known competing financial interests or personal relationships that could have appeared to influence the work reported in this study.

## DATA AVAILABILITY STATMENT

The data that support the findings of this study are available from the corresponding author upon reasonable request.

## References

[fsn33000-bib-0001] Agrawal, H. , Joshi, R. , & Gupta, M. (2016). Isolation, purification and characterization of antioxidative peptide of pearl millet (*Pennisetum glaucum*) protein hydrolysate. Food Chemistry, 204, 365–372. 10.1016/j.foodchem.2016.02.127 26988514

[fsn33000-bib-0002] Aguilar‐Toala, J. E. , & Liceaga, A. M. (2020). Cellular antioxidant effect of bioactive peptides and molecular mechanisms underlying: Beyond chemical properties. International Journal of Food Science and Technology, 56, 2193–2204. 10.1111/ijfs.14855

[fsn33000-bib-0003] Bollati, C. , Cruz‐Chamorro, I. , Aiello, G. , Li, J. Q. , Bartolomei, M. , Santos‐Sanchez, G. , Ranaldi, G. , Ferruzza, S. , Sambuy, Y. , Arnoldi, A. , & Lammi, C. (2022). Investigation of the intestinal trans‐epithelial transport and antioxidant acitivity of two hempseed peptides WVSPLAGRT (H2) and IGFLIWV (H3). Food Research International, 152, 110720. 10.1016/j.foodres.2021.110720 35181114

[fsn33000-bib-0004] Bouzenna, H. , Hfajedh, N. , Giroux‐Metges, M. A. , Elfeki, A. , & Talarmin, H. (2017). Biological properties of citral and its potential protective effects against cytotoxicity caused by aspirin in the IEC‐6 cells. Biomedicine & Pharmacotherapy, 87, 653–660. 10.1016/j.biopha.2016.12.104 28088731

[fsn33000-bib-0005] Chalamaiah, M. , Kumar, B. D. , Hemalatha, R. , & Jyothirmayi, T. (2012). Fish protein hydrolysates: Proximate composition, amino acid composition, antioxidant activities and applications: A review. Food Chemistry, 135, 3020–3038. 10.1016/j.foodchem.2012.06.100 22980905

[fsn33000-bib-0006] Chen, J. Y. , Yan, Y. J. , Zhang, L. L. , Zheng, J. Y. , Guo, J. T. , Li, R. H. , & Zeng, J. Y. (2021). Purification of novel antioxidant peptides from myofibrillar protein hydrolysate of chicken breast and their antioxidant potential in chemical and H_2_O_2_‐stressed cell systems. Food & Function, 12, 4897–4908. 10.1039/D1FO00579K 34100502

[fsn33000-bib-0007] Dai, C. , Dai, L. , Yu, F. J. , Li, X. N. , Wang, G. X. , Chen, J. , Wang, C. , & Lu, Y. P. (2020). Chemical and biological characteristics of hydrolysate of crucian carp swim bladder: Focus on preventing ulcerative colitis. Journal of Functional Foods, 75, 104256. 10.1016/j.jff.2020.104256

[fsn33000-bib-0008] Di Marzo, N. , Chisci, E. , & Giovannoni, R. (2018). The role of hydrogen peroxide in redox‐dependent signaling: Homeostatic and pathological responses in mammalian cells. Cell, 7, 156. 10.3390/cells7100156 PMC6211135 30287799

[fsn33000-bib-0009] Ghanbari, R. (2019). Review on the bioactive peptides from marine sources: Indication for health effects. International Journal of Peptide Research and Therapeutics, 25, 1187–1199. 10.1007/s10989-018-9766-x

[fsn33000-bib-0010] Ghassem, M. , Arihara, K. , Mohammadi, S. , Sani, N. A. , & Babji, A. S. (2017). Identification of two novel antioxidant peptides from edible bird's nest (*Aerodramus fuciphagus*) protein hydrolysates. Food & Function, 8, 2046–2052. 10.1039/C6FO01615D 28497137

[fsn33000-bib-0011] Jia, Q. , Yuan, J. F. , Liu, H. P. , Li, M. Y. , & Wu, Y. R. (2020). Purification and identification of dual‐enzyme hydrolysates obtained from defatted walnut and its antioxidant effects on D‐galactose‐induced aging mice. Journal of Food Measurement and Characterization, 15, 1034–1043. 10.1007/s11694-020-00702-y

[fsn33000-bib-0012] Lan, C. , Zhao, Y. Q. , Li, X. R. , & Wang, B. (2019). High Fischer ratio oligopeptides determination from Antartic krill: Preparation, peptides profiles, and in vitro antioxidant activity. Journal of Food Biochemistry, 43, e12827. 10.1111/jfbc.12827 31353526

[fsn33000-bib-0013] Li, G. S. , Zhan, J. Q. , Hu, L. P. , Yuan, C. H. , Takaki, K. , Ying, X. G. , & Hu, Y. Q. (2021). Identification of a new antioxidant peptide from porcine plasma by in vitro digestion and its cytoprotective effect on H_2_O_2_ induced HepG2 model. Journal of Functional Foods, 86, 104679. 10.1016/j.jff.2021.104679

[fsn33000-bib-0014] Luo, Q. H. , Wang, W. J. , Li, Z. , Zhu, X. H. , Wang, X. , Zhang, T. H. , Xu, H. , & Yang, J. N. (2021). Effects of diet on the volatile flavor and nutritional ingredients of common octopus (*Octopus vulgaris*). Journal of Ocean University of China, 20, 393–401. 10.1007/s11802-021-4538-1

[fsn33000-bib-0015] Mendis, E. , Rajapakse, N. , Byun, H. G. , & Kim, S. K. (2005). Investigation of jumbo squid (*Dosidicus gigas*) skin gelatin peptides for their *in vitro* antioxidant effects. Life Sciences, 77, 2166–2178. 10.1016/j.lfs.2005.03.016 15916780

[fsn33000-bib-0016] Mirdamadi, S. , Mirzaei, M. , Soleymanzadeh, N. , Safavi, M. , Bakhtiari, N. , & Zandi, M. (2021). Antioxidant and cytoprotective effects of synthetic peptides identified from *Kluyveromyces marxianus* protein hydrolysate: Insight into the molecular mechanism. LWT‐Food Science and Technology, 148, 111792. 10.1016/j.lwt.2021.111792

[fsn33000-bib-0017] Nielsen, P. M. , Petersen, D. , & Dambmann, C. (2001). Improved method for determining food protein degree of hydrolysis. Journal of Food Science, 66, 642–646. 10.1111/j.1365-2621.2001.tb04614.x

[fsn33000-bib-0018] Nordberg, J. , & Arner, E. S. J. (2001). Reactive oxygen species, antioxidants, and the mammalian thioredoxin system. Free Radical Biology & Medicine, 31, 1287–1312. 10.1016/S0891-5849(01)00724-9 11728801

[fsn33000-bib-0019] Ren, H. T. , Meng, Q. H. , Yepuri, N. , Du, X. J. , Sarpong, J. O. , & Cooney, R. N. (2018). Protective effects of glutathione on oxidative injury induced by hydrogen peroxide in intestinal epithelial cells. Journal of Surgical Research, 222, 39–47. 10.1016/j.jss.2017.09.041 29273374

[fsn33000-bib-0020] Sarmadi, B. H. , & Ismail, A. (2010). Antioxidative peptides from food proteins: A review. Peptides, 31, 1949–1956. 10.1016/j.peptides.2010.06.020 20600423

[fsn33000-bib-0021] Sila, A. , & Bougatef, A. (2016). Antioxidant peptides from marine by‐products: Isolation, identification and application in food systems: A review. Journal of Functional Foods, 21, 10–26. 10.1016/j.jff.2015.11.007

[fsn33000-bib-0022] Slama‐Ben Salem, R. , Bkhairia, I. , Abdelhedi, O. , & Nasri, M. (2017). Octopus vulgaris protein hydrolysates: Characterization, antioxidant and functional properties. Journal of Food Science and Technology, 54, 1442–1454. 10.1007/s13197-017-2567-y 28559603 PMC5430175

[fsn33000-bib-0023] Sudhakar, S. , & Nazeer, R. A. (2017). In vitro preparation and assessment of radical reducing peptide from *Octopus aegina* using digestive proteases. Journal of Bioscience and Bioengineering, 124, 36–42. 10.1016/j.jbiosc.2017.02.014 28319020

[fsn33000-bib-0024] Tkaczewska, J. , Bukowski, M. , & Mak, P. (2019). Identification of antioxidant peptides in enzymatic hydrolysates of carp (*Cyprinus Carpio*) skin gelatin. Molecules, 24, 97. 10.3390/molecules24010097 PMC6337244 30597854

[fsn33000-bib-0025] Tonolo, F. , Sandre, M. , Ferro, S. , Folda, A. , Scalcon, V. , Scutari, G. , Feller, E. , Marin, O. , Bindoli, A. , & Rigobello, M. P. (2018). Milk‐derived bioactive peptides protect against oxidative stress in Caco‐2 cell model. Food & Function, 9, 1245–1253. 10.1039/C7FO01646H 29387856

[fsn33000-bib-0026] Um, J. H. , Kim, E. A. , Lee, W. , Kang, N. , Han, E. J. , Oh, J. Y. , Park, S. Y. , Jeon, Y. J. , Lee, S. H. , & Ahn, G. (2017). Protective effects of an enzymatic hydrolysate from *Octopus ocellatus* meat against hydrogen peroxide‐induced oxidative stress in chang liver cells and zebrafish embryo. Advances in Experimental Medicine and Biology, 975, 603–620. 28849485 10.1007/978-94-024-1079-2_47

[fsn33000-bib-0027] Vaz‐Pires, P. , Seixas, P. , & Barbosa, A. (2004). Aquaculture potential of the common octopus (*Octopus vulgaris Cuvie*r, 1797): A review. Aquaculture, 238, 221–238. 10.1016/j.aquaculture.2004.05.018

[fsn33000-bib-0028] Wang, L. F. , Shi, Z. X. , Wang, X. Y. , Mu, S. , Xu, X. Y. , Shen, L. , & Li, P. (2021). Protective effects of bovine milk exosomes against oxidative stress in IEC‐6 cells. European Journal of Nutrition, 60, 317–327. 10.1007/s00394-020-02242-z 32328746

[fsn33000-bib-0029] Wang, L. Y. , Ding, L. , Yu, Z. P. , Zhang, T. , Ma, S. , & Liu, J. B. (2016). Intracellular ROS scavenging and antioxidant enzyme regulating capacities of corn gluten meal‐derived antioxidant peptides in HepG2 cells. Food Research International, 90, 33–41. 10.1016/j.foodres.2016.10.023 29195889

[fsn33000-bib-0030] Wattanasiritham, L. , Theerakulkait, C. , Wickramasekara, S. , Claudia, S. M. , & Stevens, J. F. (2016). Isolation and identification of antioxidant peptides from enzymatically hydrolyzed rice bran protein. Food Chemistry, 192, 156–162. 10.1016/j.foodchem.2015.06.057 26304333

[fsn33000-bib-0031] Wen, C. T. , Zhang, J. X. , Zhang, H. H. , Duan, Y. Q. , & Ma, H. L. (2020). Plant protein‐derived antioxidant peptides: Isolation, identification, mechanism of action and application in food systems: A review. Trends in Food Science & Technology, 105, 308–322. 10.1016/j.tifs.2020.09.019

[fsn33000-bib-0032] Wiriyaphan, C. , Xiao, H. , Decker, E. A. , & Yongsawatdigul, J. (2015). Chemical and cellular antioxidative properties of threadfin bream (*Nemipterus spp*.) surimi byproduct hydrolysates fractionated by ultrafiltration. Food Chemistry, 167, 7–15. 10.1016/j.foodchem.2014.06.077 25148952

[fsn33000-bib-0033] Wu, R. B. , Huang, J. F. , Huan, R. , Chen, L. L. , Yi, C. P. , Liu, D. , Wang, M. , Liu, C. L. , & He, H. L. (2021). New insights into the structure‐activity relationships of antioxidative peptide PMRGGGGYHY. Food Chemistry, 337, 127678. 10.1016/j.foodchem.2020.127678 32791429

[fsn33000-bib-0034] Wu, R. B. , Wu, C. L. , Liu, D. , Yang, X. H. , Huang, J. F. , Zhang, J. , Liao, B. Q. , & He, H. L. (2018). Antioxidant and anti‐freezing peptides from salmon collagen hydrolysate prepared by bacterial extracellular protease. Food Chemistry, 248, 346–352. 10.1016/j.foodchem.2017.12.035 29329864

[fsn33000-bib-0035] Yang, J. , Huang, J. C. , Dong, X. L. , Zhang, Y. L. , Zhou, X. H. , Huang, M. , & Zhou, G. H. (2020). Purification and identification of antioxidant peptides from duck plasma proteins. Food Chemistry, 319, 126534. 10.1016/j.foodchem.2020.126534 32193058

[fsn33000-bib-0036] Yi, J. E. , Zhao, J. , & Wu, J. P. (2017). Egg ovotransferrin derived IRW exerts protective effect against H_2_O_2_‐induced oxidative stress in Caco‐2 cells. Journal of Functional Foods, 39, 160–167. 10.1016/j.jff.2017.10.012

[fsn33000-bib-0037] Zhang, C. , Zhang, Y. X. , Xia, S. Q. , Zhu, S. Y. , Li, H. , & Liu, X. Q. (2021). Evaluating the effects of MKAVCFSL derived from bighead carp (*Hypophthalmichthys nobilis*) flesh on antioxidant activity in Caco‐2 cells in vitro. Journal of Food Quality, 2021, 9975586. 10.1155/2021/9975586

[fsn33000-bib-0038] Zhang, H. S. , Xue, J. , Zhao, H. X. , Zhao, X. S. , Xue, H. H. , Sun, Y. H. , & Xue, W. R. (2018). Isolation and structural characterization of antioxidant peptides from degreased apricot seed kernels. Journal of AOAC International, 101, 1661–1664. 10.5740/jaoacint.17-0465 29724259

[fsn33000-bib-0039] Zhang, Q. Z. , Tong, X. H. , Li, Y. , Wang, H. , Wang, Z. J. , Qi, B. K. , Sui, X. N. , & Jiang, L. Z. (2019). Purification and characterization of antioxidant peptides from alcalase‐hydrolyzed soybean (*Glycine max L*.) hydrolysate and their cytoprotective effects in human intestinal Caco‐2 cells. Journal of Agricultural and Food Chemistry, 67, 5772–5781. 10.1021/acs.jafc.9b01235 31046268

[fsn33000-bib-0040] Zhong, H. , Abdullah , Zhang, Y. P. , Deng, L. L. , Zhao, M. J. , Tang, J. , Zhang, H. , Feng, F. Q. , & Wang, J. (2021). Exploring the potential of novel xanthine oxidase inhibitory peptide (ACECD) derived from Skipjack tuna hydrolysates using affinity‐ultrafiltration coupled with HPLC‐MALDI‐TOF/TOF‐MS. Food Chemistry, 347, 129068. 10.1016/j.foodchem.2021.129068 33486365

[fsn33000-bib-0041] Zou, T. B. , He, T. P. , Li, H. B. , Tang, H. W. , & Xia, E. Q. (2016). The structure‐activity relationship of the antioxidant peptides from natural proteins. Molecules, 21, 72. 10.3390/molecules21010072 26771594 PMC6273900

